# Drinking Over the Lifespan: Focus on Early Adolescents and Youth

**DOI:** 10.35946/arcr.v38.1.12

**Published:** 2016

**Authors:** Michael Windle

**Affiliations:** Michael Windle, Ph.D., is a professor in the Department of Behavioral Sciences and Health Education, Emory University, Atlanta, Georgia

**Keywords:** Alcohol consumption, alcohol use frequency, alcohol use pattern, alcohol-related problems, alcohol misuse, alcohol sensitivity, binge drinking, heavy drinking, adolescent drinking, underage drinking, adolescent, youth, alcohol and other drug use, alcohol-related traffic fatalities, sexuality, risky sexual behavior, academic performance, risk factors, brain development, growth and development

## Abstract

Historical trends in alcohol use among U.S. adolescents, as well as data regarding alcohol-related traffic fatalities among youth, indicate decreases in alcohol use. Nevertheless, alcohol use patterns still indicate high rates of binge drinking and drunkenness and the co-occurrence of alcohol use among youth with risky sexual activity, illicit substance use, and poor school performance. This article discusses unique elements of alcohol use among adolescents relative to adults that pose risks for alcohol misuse and alcohol-related problems. These differences range from patterns of drinking to differential sensitivity to alcohol. Developmental differences between adolescents and adults also are discussed with regard to age-normative developmental tasks and distinctions in brain development that may affect differences in drinking patterns. Epidemiologic findings on sexual-minority youth are provided, as are global trends in alcohol use among early adolescents and youth. It is proposed that using information about differences between youth and adults will be helpful in directing future etiologic and intervention research by capitalizing on unique biological, psychological, and social factors that may affect the success of efforts to reduce alcohol use among early adolescents and youth.

In describing patterns of alcohol use among early adolescents (ages 12–14) and youth (ages 15–20), there is both good news and bad news. The good news is that research findings with U.S. national epidemiology data from long-term annual surveys of high-school students, such as the Monitoring the Future surveys, have indicated historical shifts toward overall decreases in levels of alcohol use among early adolescents and youth (Johnston et al. 2013). For example, national data from the Monitoring the Future studies have indicated that in 2012, historic lows in the prevalence of alcohol use were reported across all three grade levels assessed (i.e., 8th, 10th, and 12th graders). Self-reported alcohol use in the prior 30 days for the three respective grade levels were 11 percent, 28 percent, and 41 percent. By contrast, in 2000, these respective last 30-day prevalence rates were 22 percent, 41 percent, and 50 percent. Consistent with these findings are those reported in the National Household Survey on Drug Use and Health that indicate decreases in heavy episodic or binge drinking for birth cohorts born in the 1990s relative to birth cohorts born previously, going back as far as the 1950s (Keyes and Miech 2013). The number of drinking-and-driving traffic fatalities involving 16- to 20-year-olds also has decreased from 5,244 in 1982 (which accounted for 66 percent of traffic fatalities) to 1,262 in 2010 (which corresponded to 37 percent of traffic fatalities) (Hingson and White 2014; Voas et al. 2012). These U.S. national epidemiologic findings are encouraging in that the historical trends indicate decreases and, in some instances (e.g., traffic fatalities), substantial decreases in alcohol use and adverse consequences among young people.

The bad news is despite these reductions in the overall prevalence of alcohol use among early adolescents and youth, alcohol remains the substance of choice among early adolescents and youth and still is used by a majority of youth. The table summarizes 2012 U.S. national findings for alcohol use from the Monitoring the Future survey (Johnston et al. 2013). Although historically the prevalence of alcohol use may be decreasing, the rates still are quite high for heavier use, with almost one-quarter of 12th graders reporting binge drinking (i.e., 5 or more drinks in a row in the past 2 weeks) and almost one-half of 12th graders and more than one-quarter of 10th graders reporting being drunk in the past 12 months. The prevalence of any use in the past year also remained high for 10th and 12th graders, and almost one-quarter of 8th graders reported past-year use. The findings in the table also indicate the high prevalence of using flavored alcohol beverages, especially among 8th graders. The ratio (translated to a percentage) of using any flavored alcohol beverages in the past 30 days and any use of alcohol in the past 30 days was 52 percent for 12th graders, 59 percent for 10th graders, and 69 percent for 8th graders. Hence, relatively new alcohol products in the market place, such as those with sweet flavoring, seem to be among the alcohol beverages of choice, especially among early adolescents (i.e., 8th graders), although high rates also were reported by 10th and 12th graders.

Gender differences for the alcohol use indicators in the table tend to reflect a high degree of convergence across sexes, especially among 8th and 10th graders. Among 8th graders, girls reported higher levels than boys for the prevalence of alcohol use during the past 12 months and past 30 days, a higher prevalence of using flavored alcohol beverages, and a higher prevalence of binge drinking in the past 2 weeks and having been drunk in the past 12 months. Among 10th graders, the prevalence across alcohol indicators is similar across gender groups, with the exception that girls used more flavored alcohol beverages. Among 12th graders, a higher percentage of boys than girls reported engaging in heavier drinking (i.e., binge drinking in the past 2 weeks and having been drunk in past 12 months). Race/ethnic group differences indicated that for 8th graders, the prevalence of the alcohol use indicators was lower for Whites and African Americans but higher for Hispanics. By contrast, for 10th and 12th graders, Whites and Hispanics reported a higher prevalence of the alcohol use indicators than African Americans. These findings are similar to prior years of the Monitoring the Future surveys in indicating a lower prevalence of alcohol use among African American early adolescents and youth and a higher prevalence among White and Hispanic youth. Similar ethnic group findings also were reported for the 2013 Youth Risk Behavior Surveillance Survey (Kann et al. 2014).

Alcohol use by early adolescents and youth also remains highly correlated with a range of other risky behaviors, including tobacco use, co-occurring illicit substance use, sexually risky behaviors (e.g., unprotected sex), lower school performance, and externalizing behavioral problems (e.g., conduct problems, delinquency), as well as with the three highest manifestations of adolescent mortality (drinking- and-driving crashes, suicide, and homicide) (Epstein et al. 2013). Furthermore, although substantial strides have been made in reducing alcohol-related traffic fatalities among youth, national findings for 9th through 12th graders from the 2011 Youth Risk Behavioral Survey indicated that the prevalence of drinking and driving in the past 30 days was 8.2 percent and riding with a drinking driver was 24.1 percent (Eaton et al. 2012). Hence, the good news described previously must be tempered with a more comprehensive evaluation of the available data and recognition that many challenges regarding alcohol use among early adolescents and youth remain to be addressed. (See the accompanying sidebar for a brief review of emerging risks from greater access to marijuana and alternative tobacco products, which may interact with adolescent and youth alcohol use.)

This article reviews several issues related to early adolescent and youth alcohol use to further illuminate why this period of development in the lifespan remains of high importance and is a special population. Note that across the research literature a range of definitions are used to describe the adolescent phase in the lifespan. In this article, we use the term early adolescence to refer to the age range 12 through 14 (which includes, among others, 8th graders) and youth for the age range 15 through 20; adolescence refers to the entire age range of 12- to 20-year-olds. This article highlights four areas to demonstrate how this period of the lifespan differs from others with regard to alcohol use, its consequences, and the implications for prevention and treatment. The first part examines differences in alcohol use patterns and sensitivity to alcohol for early adolescents and youth relative to adults. Second, differences between early adolescents and youth and adults are discussed with regard to differences in development, with particular reference to age-normative psychosocial tasks (e.g., puberty, friendship formation) and to brain development that uniquely occurs during this phase of the lifespan. The third section discusses findings regarding alcohol use among sexual minority youth. Fourth, global patterns of alcohol use among early adolescents and youth are presented.

## Alcohol Use Patterns and Sensitivity to Alcohol

National household data reveal distinct differences in patterns of alcohol use between early adolescents and youth and adults with regard to number of drinking days per month and usual number of drinks per occasion (Substance Abuse and Mental Health Services Administration [SAMHSA] 2006). Adolescents (summed across ages 12–20) reported 6 drinking days per month on average, whereas young adults (ages 21–25) reported an average of 8 days per month, and adults (ages 26 or older) reported almost 9 days per month. However, adolescents reported an average of 5 drinks per occasion, compared with 4 drinks for young adults and 3 drinks for adults. Hence, drinking among adolescents is less frequent than for adults, but the amount consumed per occasion is considerably more, with average levels of drinking that meet criteria for binge or heavy episodic drinking. Although there is some variation in the definition of binge or heavy episodic drinking it often is defined as at least 5 drinks in a row in the past 2 weeks (Johnston et al. 2013). Adolescents tend to consume alcohol much more opportunistically than adults (e.g., at parties), and heavy drinking is the norm rather than exception when these opportunities arise. Of course, there are situations when adults also drink heavily, but the more general pattern is one of frequent drinking at lower levels. A major issue with the higher frequency of heavier drinking among adolescents is that it may contribute to a broad range of co-occurring problems (e.g., sexual or physical victimization, drinking and driving) that may have short- and long-term adverse consequences.

Adolescents and adults also differ in their sensitivity to the effects of alcohol. Because administering alcohol to human adolescents is fraught with ethical and legal challenges, rats are often used to compare adolescents and adults while experimentally manipulating other variables, such as levels of alcohol exposure. Research findings with rats have indicated that following the administration of ethanol, adolescent rats performed more poorly than adult rats on a range of memory and learning discrimination tasks, thereby suggesting a greater sensitivity to alcohol as manifested by impaired memory performance during adolescence (for review, see Spear and Swartzwelder 2014). Other research comparing adolescent and adult rats has indicated that adolescent rats have reduced sensitivity to alcohol’s aversive and undesirable consequences, such as motor impairment and drowsiness (i.e., sedative effects) (Spear and Swartzwelder 2014).

Hence, these comparative studies of adolescent and adult rats, along with human studies that indicate memory differences between younger (early 20s) and somewhat older (late 20s) adults (Acheson et al. 1998), suggest adolescents and adults may manifest differential sensitivity in their acute responses to alcohol. These differential sensitivities may affect patterns and consequences of drinking for these age-groups. For example, if youth are not experiencing the sedative effects of alcohol, they may continue to drink rather than reduce or stop their drinking. Similarly, if learning and memory are impaired as a result of alcohol use, decision making may likewise be impaired and current, immediate situational cues or determinants (e.g., drinking and driving with friends) may override more reflective cognitive processes that, in the absence of alcohol consumption, could lead to less risky behaviors. Caution needs to be exerted in drawing conclusions for human behaviors based on animal model findings. Whereas animal and some human studies have suggested age differences in response to alcohol, other studies have suggested similarities between human adolescents and adults with regard to some biochemical parameters (e.g., serum acetate concentration) related to alcohol intoxication (Lamminpaa 1995). Nevertheless, the notion of age-related differential sensitivity to alcohol remains a vibrant area of research.

## Developmental Tasks and Brain Development

A number of developmental theorists, including Erik Erickson (1950), have postulated that people confront different age-appropriate developmental tasks during phases or periods of the lifespan. The period of adolescence is characterized by a host of developmental changes and challenges, including puberty; significant increases in physical size and changes in physical appearance; confrontation with issues of personal, ethnic, and sexual identity; renegotiating relationships with parents toward a greater acceptance of personal autonomy; becoming more peer involved and influenced; initiating and maintaining dating relationships; and changing schools. These developmental changes and challenges occur during a period of life that intersects with the onset and escalation of alcohol and other substance use and can pose unique risks for adolescents. For instance, early pubertal development by girls may increase their risk of early-onset alcohol use through their involvement with older boys (Lanza and Collins 2002). Similarly, greater affiliation with peers can yield both positive benefits (e.g., increased social skills) and negative costs if alcohol use becomes a dominant element of friendship groups. The intersection between challenging developmental tasks and alcohol use across adolescence was a significant topic associated with the NIAAA Underage Drinking Initiative (2014), and a special supplemental issue of *Pediatrics* provides an expanded discussion of these issues (see Brown et al. 2008; Windle et al. 2008).

Brain development represents another significant area of change that occurs during adolescence. Research has focused on how brain development may influence adolescent alcohol use and vice versa—that is, how alcohol use may influence the developing brain. Although overall brain size achieves its peak in early childhood, maturational changes in brain cortical volume, axonal growth, and refinement of cortical connections (e.g., via synaptic “pruning”) continue, especially with regard to the limbic system, including the amygdala and the prefrontal cortex (Bava and Talpert 2010). These brain systems are involved in a broad range of cognitive, affective, and behavioral processes (e.g., learning, decision making, impulsivity) that, in turn, influence alcohol use and other co-occurring problems (e.g., risky sexual behavior).

With regard to the maturation of the brain, important developmental asynchronies exist between some earlier developing limbic and affective portions of the brain relative to the later developing prefrontal cortex. This is significant because in earlier adolescence, the affective portions of the brain may be more dominant with respect to behavioral responses, including the immediate rewarding aspects of alcohol use, whereas the brain functions associated with the prefrontal cortex that involve higher cognitive processing related to executive functioning (e.g., planning, goal setting, inhibitory control), decision making, and cognitive-affective behavioral regulation still are developing. Hence, metaphorically, in earlier adolescence the dominant affective system says “Go” (e.g., drink alcohol for immediate reward) without the counteracting effect of the later-to-develop “Stop” system associated with brain functions of the prefrontal cortex.

The asynchrony described above is unique during adolescence and dovetails with research on reward-seeking behavior among adolescents and reward sensitivity in the dopamine-rich brain striatum (Galvan 2010). This research indicates that during adolescence, an increased activation in reward sensitive areas of the brain contributes to adolescents seeking, or being highly motivated to pursue, appetitive rewards (e.g., alcohol). The neuroscience research indicates that this phenomenon of heightened sensitivity to reward is unique to adolescence and does not occur in childhood or adulthood (Galvan 2010; Spear 2011). Spear summarized research conducted with adolescent rats supporting not only heightened sensitivity to the rewarding effects of alcohol but also to the facilitation of social behavior by alcohol, thereby contributing to rewarding effects of alcohol in social contexts. She further proposed that such reward-oriented propensities during adolescence may contribute to adolescents’ differential evaluation of the costs and benefits of alcohol use compared with individuals at other ages (i.e., adolescents would estimate that alcohol use has greater benefits and fewer costs). Hence, the existing neuroscience literature is contributing to a more nuanced understanding of why adolescence is a unique period of development and is identifying cognitive (e.g., impulsive decision making) and affective (e.g., heightened reactivity) mechanisms that may serve as targets for intervention and/or provide clarity for components of intervention programs (Riggs and Greenberg 2009).

## Alcohol Use Among Adolescent Sexual-Minority Youth

Sexual orientation and the development of a sexual identity become especially prominent following puberty, with the occurrence of developmental tasks related to establishing a sense of personal and sexual identity, peer selection and socialization, and the initiation and escalation of romantic relationships. Relative to the longer-term study of sexual orientation and alcohol use among adults, large-scale epidemiologic findings of adolescent sexual orientation and alcohol use have a relatively brief history. Nevertheless, several recent studies have yielded consistent findings with regard to sexual orientation and substance use, including alcohol use. Marshal and colleagues (2008) conducted a meta-analysis of existing studies on adolescent sexual orientation and substance use and reported that lesbian, gay, and bisexual (LGB) youth reported substance use at almost twice the rate of heterosexual youth and that subgroups at particularly high risk were bisexuals and sexual-minority females. Youth who identified as “mostly heterosexual” also used substances at levels similar to bisexual youth. Talley and colleagues (2014) used data from the 2005 and 2008 Youth Risk Behavior Surveys and reported similar findings—sexual-minority youth (i.e., those who self-identified as not exclusively heterosexual) reported higher rates of alcohol use than their heterosexual counterparts. Of particular interest, the age of drinking onset for sexual-minority youth indicated that 35.6 percent had initiated use at or before age 12, relative to 21.7 percent of heterosexuals. The rate of past-month heavy episodic drinking was almost twice as high among sexual-minority youth, as was the number of past-month drinking days, using a cut point of 6 or more days. Bisexual youth, sexual-minority females, and younger sexual-minority youth reported the highest rates of alcohol use.

In addition to these cross-sectional epidemiologic findings indicating higher levels of alcohol and other substance use among sexual-minority youth relative to exclusively heterosexual youth, two studies have used longitudinal data from the National Longitudinal Study of Adolescent Health (AddHealth) to investigate alcohol and substance use outcomes in young adulthood. Marshal and colleagues (2009) used three-wave data (at wave 1 average age about 15.8 years, then a 1-year followup, and an additional 5-year followup). Nonexclusively heterosexual orientation predicted more rapid increases in substance use, including alcohol use and drunkenness, across this adolescent to early young adulthood period. Dermody and colleagues (2014) extended this time window by using four waves of AddHealth data (ages 14–18 at wave 1; 27–31 years at wave 4) to study associations between self-identified sexual-minority youth and exclusively heterosexual youth on a measure of hazardous drinking, defined by frequency of drunkenness. The longitudinal findings indicated that in later young adulthood (ages 27–31), sexual-minority youth, as self-identified during adolescence, had significantly higher levels of hazardous drinking than heterosexual youth and that the magnitude of these differences increased across time, especially among men. Some mechanisms have been proposed for the higher rates of alcohol and other substance use among sexual-minority youth, such as stress-related stigmatization that may contribute to stress-relief drinking (Hatzenbuehler et al. 2008). Nevertheless, considerable research remains to be completed on identifying the underlying mechanisms for a higher prevalence of alcohol use among this sexual-minority population and using this information to guide preventive interventions.

**Alcohol Use, Emerging Tobacco Products, and Marijuana Use**

Among early adolescents and youth, alcohol use commonly co-occurs with other substance use and problem behaviors (Biglan et al. 2004). Furthermore, with new alcohol and tobacco products being created and marketed to early adolescents and youth, the field of alcohol research must consider the impact of recent historical events and trends manifested in related areas of substance use. Two contemporary trends immediately come to mind.

First, medical marijuana use, along with marijuana de-criminalization and marijuana legalization in some States (the latter in Colorado and Washington), may have an impact on rates of alcohol use, co-use of alcohol and emerging tobacco products, and increased polydrug use of alcohol, marijuana, and tobacco or nicotine-based products. The social norms for the perceived dangers and social acceptability of marijuana use among youth already are shifting toward reduced harm and greater social acceptance (Johnston et al. 2013).

Second, the availability and prevalence of alternative and emerging tobacco products (e.g., snus, cigars, cigarillos, hookah, electronic cigarettes [e-cigarettes]) is increasing. Nicotine use may re-emerge as a prominent gateway substance that fosters further escalation and continued drug use. The advertising associated with many of these alternative tobacco products also is geared toward youth, with flavors (e.g., cherry, cinnamon) reminiscent of the “alcopop” (flavored alcohol beverages) industry and its marketing efforts that target young people. Annual high school surveys from 2007 to 2012 in the State of Florida indicated significant decreases in cigarette use, largely offset by increases in alternative tobacco products (Barnett et al. 2014). The Food and Drug Administration currently is considering regulations for e-cigarettes that contain nicotine but not tar or many of the other carcinogens of tobacco. However, with the current unregulated state of e-cigarettes, nicotine levels vary widely across brands and may undermine arguments for their use as a harm-reduction product.

More research is needed to determine the potential benefits and costs of e-cigarettes. But their rapid and widespread use, along with other alternative tobacco products, in conjunction with a more permissive attitude toward marijuana use, may make it more difficult to prevent co-use and polydrug use patterns. Alternative tobacco products may affect attempts to prevent the onset and escalation of alcohol use, as well as relapse among those treated for alcohol disorders.

## Global Patterns of Alcohol Use Among Early Adolescents and Youth

Alcohol use and its adverse consequences among early adolescents and youth have become of increasing interest on the global stage. Gore and colleagues (2011) reported on youth alcohol use in the World Health Organization (WHO) Global Burden of Disease study. This worldwide study derived an index of disability-adjusted life-years (DALYs) for all participants by combining years of life lost because of premature mortality and years of life lost as a result of incident cases of disease or injury. One DALY corresponds to the loss of 1 year of full health. Among youth aged 10–24, the main risk factor for incident DALYs was alcohol use, which accounted for 7 percent of the DALYs. Alcohol was the most prominent risk factor worldwide for 15- to 24-year-olds. Unsafe sex, which often co-occurs with alcohol use, was the second highest risk factor, accounting for 4 percent of the incident DALYs. Gore and colleagues (2011) concluded that many risk factors and noncommunicable diseases, such as alcohol use and alcohol disorders, other psychiatric disorders, and injury, often have not been prioritized by the global public health community and that data such as these findings with youth suggest that a higher priority would be beneficial.

Findings reported in the WHO Health Behavior in School Children (HBSC) study (Currie et al. 2008), an investigation of multiple health behaviors, including alcohol use, across 23 European countries and North American countries, also indicated global patterns of alcohol use among early adolescents and youth. For example, findings from the HBSC using drunkenness as a measure of alcohol misuse indicated a significant increase in drunkenness from ages 11–15, with the steepest increase from ages 13–15; boys reported a higher prevalence of drunkenness than girls across almost all countries, and the prevalence of drunkenness was higher in northern than southern Europe (Currie et al. 2008). A more detailed analysis of changes in adolescent drunkenness from HBSC surveys in 1997–1998 and 2005–2006 indicated a significant decrease (25 percent on average) in adolescent drunkenness among 13 of 16 Western countries but a significant increase (40 percent in mean frequency) in adolescent drunkenness in 7 Eastern European countries (Kuntsche et al. 2011). A more in-depth presentation of the HBSC surveys is beyond the scope of this article, but more extensive findings on cross-national comparisons are provided by Bendtsen and colleagues (2014), and findings specifically on U.S. national data for 6th through 10th graders who participated in the HSBC study are provided by Brooks-Russell and colleagues (2014).

With increasing economic and cultural globalization, alcohol use often is increasing in developing countries where the prevalence of drinking was previously relatively low. For example, Prasad (2009) reported that sales of alcohol in India grew by 8 percent in the previous 3 years. This is thought to be a serious underestimate, because almost two-thirds of the alcohol consumed in India is not recorded (e.g., local home brew, or smuggled into the country). Of particular concern with regard to youth is that drinking alcohol is becoming more prevalent among younger people (under age 21), with rates increasing from 2 percent to 14 percent over the past 15 years. Furthermore, at the national level in India, alcohol-related problems account for 20 percent of hospital admissions, 18 percent of psychiatric admissions, 20 percent of brain injuries, and 60 percent of all injuries in India’s emergency rooms. Although these statistics are for all patients, not just early adolescents and youth, they forebode unhealthy outcomes for this population, especially given the recent historical trends toward increases in alcohol use at earlier ages.

## Conclusions

Recent U.S. historical trends regarding alcohol use among early adolescents and youth have indicated significant reductions in use that have been paralleled by substantial reductions in alcohol-related traffic fatalities among youth. These trends are positive and suggest that our efforts to modify early adolescent and youth drinking through intervention programs and alcohol policies are yielding valuable gains. Nevertheless, the epidemiologic data still indicate serious problems with alcohol use among early adolescents and youth, with the prevalence of binge drinking, drunkenness, drinking and driving, and driving with someone who has been drinking still at high levels. Sexual-minority youth are at particularly high risk for alcohol misuse, and adolescence is a critical phase in development for establishing personal and sexual identity. Additional research is needed to understand the complexities involved in these higher levels of use among sexual-minority youth. It also is clear that as globalization continues, alcohol use and misuse among early adolescents and youth is becoming more pervasive and impacting youth internationally. A number of characteristics distinguish adolescent from adult drinking, including a higher number of drinks per occasion by adolescents, different sensitivities to the effects of alcohol on adolescents and adults, and developmental differences in psychosocial tasks and brain development. Applying this information about differences between adolescents and adults will be helpful in directing future etiologic and intervention research because it will facilitate a focus on unique biological, psychological, and social factors that may affect the success of efforts to reduce alcohol use among early adolescents and youth.

## Figures and Tables

**Table:** Prevalence of Alcohol Use (Percentage) by Demographic Subgroups Among 8th, 10th, and 12th Graders, 2012

	Any Use in Past 12 Months	Any Use in Past 30 Days	Flavored Alcohol Beverages in Past 30 Days	5+ Drinks in a Row in Past 2 Weeks	Been Drunk in Past 12 Months
**8th Graders**					
Total	23.6	11.0	7.6	5.1	8.6
Gender					
Boys	22.3	10.3	6.1	4.6	7.8
Girls	24.7	11.6	9.2	5.5	9.3
Race/Ethnicity					
White	23.5	10.7	7.4	4.9	9.3
African American	22.4	10.0	8.8	4.3	6.5
Hispanic	33.4	17.5	10.8	9.9	12.6

**10th Graders**					
Total	48.5	27.6	16.3	15.6	28.2
Gender					
Boys	47.8	28.0	13.5	16.4	28.5
Girls	49.2	27.1	18.5	14.8	28.1
Race/Ethnicity					
White	50.9	29.1	16.5	16.3	31.1
African American	41.0	20.2	10.7	8.2	18.6
Hispanic	51.1	28.4	17.7	17.1	28.2

**12th Graders**					
Total	63.5	41.5	21.8	23.7	45.0
Gender					
Boys	63.7	43.8	19.9	27.2	47.7
Girls	62.9	38.8	23.3	19.7	41.3
Race/Ethnicity					
White	66.3	43.8	22.9	25.7	47.5
African American	52.4	29.6	17.2	11.3	24.2
Hispanic	64.0	39.8	26.8	21.8	40.2

SOURCE: Johnston et al. 2013.
